# Trastuzumab, pertuzumab, and eribulin mesylate versus trastuzumab, pertuzumab, and a taxane as a first-line or second-line treatment for HER2-positive, locally advanced or metastatic breast cancer: study protocol for a randomized controlled, non-inferiority, phase III trial in Japan (JBCRG-M06/EMERALD)

**DOI:** 10.1186/s13063-020-04341-y

**Published:** 2020-05-07

**Authors:** Toshinari Yamashita, Norikazu Masuda, Shigehira Saji, Kazuhiro Araki, Yoshinori Ito, Toshimi Takano, Masato Takahashi, Junji Tsurutani, Kei Koizumi, Masahiro Kitada, Yasuyuki Kojima, Yasuaki Sagara, Hiroshi Tada, Tsutomu Iwasa, Takayuki Kadoya, Tsuguo Iwatani, Hiroki Hasegawa, Satoshi Morita, Shinji Ohno

**Affiliations:** 1 Department of Breast Surgery, Kanagawa Cancer Center, 2-3-2 Nakao Asahi-ku, Yokohama-shi, Kanagawa 241-8515 Japan; 2 Department of Surgery, Breast Oncology, National Hospital Organization Osaka National Hospital, 2-1-14 Hoenzaka, Chuou-ku, Osaka, 540-0006 Japan; 3 Department of Medical Oncology, Fukushima Medical University, 1 Hikarigaoka Fukushima, Fukushima, 960-1295 Japan; 4 Department of Breast Surgery, Gunma Prefectural Cancer Center, 617-1 Takahayashinishicho, Ota, Gunma 373-8550 Japan; 5 Breast Medical Oncology, Breast Oncology Center, The Cancer Institute Hospital of JFCR, 3-8-31 Ariake Koto-ku, Tokyo, 135-8550 Japan; 6 Department of Medical Oncology, Toranomon Hospital, 2-2-2 Toranomon Minato-ku, Tokyo, 105-8470 Japan; 7 Breast Surgery, NHO Hokkaido Cancer Center, 2-3-54 Yonjyo Kikusui Shiraishi-ku, Sapporo-shi, Hokkaido 003-0804 Japan; 8 Department of Medical Oncology, Showa University Hospital, 1-5-8 Hatanodai Shinagawa-ku, Tokyo, 142-8666 Japan; 9 First Department of Surgery, Hamamatsu University School of Medicine, 1-20-1 Handayama, Higashi-ku, Hamamatsu City, Shizuoka 431-3192 Japan; 10 Breast Disease Center, Asahikawa Medical University Hospital, 1-1 Higashi 2-jyo 1-chome, Midorigaoka, Asahikawa-shi, Hokkaido 078-8510 Japan; 11 Department of Breast Surgery, St. Marianna University School of Medicine Hospital, 2-16-1 Sugao Miyamae-ku, Kawasaki-shi, Kanagawa 216-8511 Japan; 12 Breast Surgical Oncology, Sagara Hospital, 3-31 Matsubaracho Kagoshima-shi, Kagoshima, 892-0833 Japan; 13 Department of Breast and Endocrine Surgical Oncology, Tohoku University Hospital, 1-1 Seiryocho Aoba-ku Sendai-shi, Miyagi, 980-8574 Japan; 14 Oncology Internal Medicine, Kindai University Hospital, 377-2 Ohnohigashi Sayama-shi Osaka, Osaka, 589-8511 Japan; 15 Breast Surgery, Hiroshima University Hospital, 1-2-3 Kasumi Minami-ku Hiroshima-shi, Hiroshima, 734-8551 Japan; 16 Department of Breast Surgery, National Cancer Center Hospital East, 6-5-1 Kashiwanoha, Kashiwa, Chiba 277-8577 Japan; 17 Eisai Co., Ltd., 4-6-10 Koishikawa Bunkyo-ku, Tokyo, 112-8088 Japan; 18 Department of Biomedical Statistics and Bioinformatics, Graduate School of Medicine Kyoto University, 54 Kawaharacho, Shogoin, Sakyo-ku, Kyoto, 606-8507 Japan; 19 Breast Oncology Center, The Cancer Institute Hospital of JFCR, 3-8-31 Ariake Koto-ku, Tokyo, 135-8550 Japan

**Keywords:** Metastatic breast cancer, HER2-positive, Non-inferiority, Eribulin, Taxane, Trastuzumab, Pertuzumab, Combination therapy

## Abstract

**Background:**

Trastuzumab (Tmab), pertuzumab (Pmab), and taxane has been a standard first-line treatment for recurrent or metastatic human epidermal growth factor (HER2)-positive breast cancer (HER2^+^ mBC) but has some safety issues due to taxane-induced toxicities. This has led to ongoing efforts to seek less toxic alternatives to taxanes that are equally effective when used in combination with Tmab plus Pmab. This study aims to show the non-inferiority of eribulin, a non-taxane microtubule inhibitor, against taxane, as a partner for dual HER2 blockade.

**Methods/design:**

This multicenter, randomized, open-label, parallel-group, phase III study will involve a total of 480 Japanese women with HER2^+^ mBC who meet the following requirements: (1) age 20–70 years; (2) no prior cytotoxic chemotherapy (excluding trastuzumab-emtansine) for mBC; (3) ≥ 6 months after prior neoadjuvant or adjuvant cytotoxic chemotherapy; (4) presence of any radiologically evaluable lesion; (5) left ventricular ejection fraction ≥ 50%; (6) Eastern Cooperative Oncology Group performance status score of 0 or 1; (7) adequate organ function; and (8) life expectancy of at least 6 months. They will be randomized 1:1 to receive eribulin (1.4 mg/m^2^ on days 1 and 8) or taxane (docetaxel 75 mg/m^2^ on day 1 or paclitaxel 80 mg/m^2^ on days 1, 8, and 15) in combination with Tmab (8 mg/kg then 6 mg/kg) plus Pmab (840 mg then 420 mg) on day 1 of each 21-day cycle. The treatment will be continued until disease progression or unmanageable toxicity. The primary endpoint is progression-free survival as per investigator according to RECIST v1.1 criteria. Key secondary endpoints include objective response rate, overall survival, quality of life and safety. Non-inferiority will be tested with two margins of 1.33 and 1.25 in a stepwise manner. If non-inferiority is shown with a margin of 1.25, superiority will then be tested.

**Discussion:**

If this study shows the non-inferiority, or even superiority, of Tmab, Pmab, and eribulin against the existing taxane-containing regimen, this new regimen may become a standard first- or second-line treatment option for HER2^+^ mBC in Japan.

**Trial registration:**

ClinicalTrials.gov, ID: NCT03264547. Registered on 28 June 2017.

## Background

In Japan, breast cancer has been the most common type of malignancy among women since 1995, with an estimated 95,000 women with newly diagnosed breast cancer during the year 2016 [1]. About 5% of new cases of breast cancer are already at advanced stages at the time of diagnosis, and about 30% of breast cancer patients experience recurrence after their initial treatment [2]. Despite the use of multi-modality treatment approaches, locally advanced or metastatic breast cancer (mBC) still has a poor prognosis and a very low rate of cure, with only about 5% of those patients expected to survive for 10 years [3].

If these patients have tumors which express human epidermal growth factor (HER2), the addition of anti-HER2 therapy is also recommended based on high-quality evidence showing survival benefits of combining anti-HER2 therapy with chemotherapy for HER2-positive (HER2^+^) mBC [4]. The current Japanese practice guidelines most strongly recommend the combination of trastuzumab (Tmab), pertuzumab (Pmab) and docetaxel (DTX) as a first-line treatment regimen for HER2^+^ mBC that is newly diagnosed or has recurred after neoadjuvant and/or adjuvant chemotherapy. These guideline recommendations of upfront Pmab for HER2^+^ mBC are based on the results of several clinical studies in this setting. These include the phase III CLEOPATRA study, which showed significantly better outcomes in patients treated with Pmab versus placebo in combination with Tmab plus DTX in terms of objective response rate (ORR; 80.2% vs 69.3%), progression-free survival (PFS; 18.5 vs 12.4 months) and overall survival (OS; 56.5 vs 40.8 months) [5, 6]. However, use of taxanes such as DTX can cause unacceptable hematologic as well as non-hematologic toxicities, including edema and peripheral neuropathy, which may result in dose reduction and/or delay and may impair the quality of life (QOL) of patients. These safety concerns have led to ongoing efforts to develop less toxic alternatives to taxanes that are equally effective when used in combination with Tmab plus Pmab.

Eribulin is a synthetic analog of halichondrin B (HalB), a substance isolated from the rare marine sponge *Halichondria okadai*. Eribulin suppresses mitosis by directly binding to microtubule ends and by inducing tubulin aggregates, which compete with unligated soluble tubulin to form additions to the ends of growing microtubules [7]. In the open-label, phase III EMBRACE study, women with heavily treated locally recurrent or mBC (16% of whom had HER2^+^ disease) were randomized to receive eribulin or treatment of physician’s choice (TPC). Compared with TPC, eribulin significantly improved OS (median: 13.1 vs 10.6 months; hazard ratio (HR) 0.81; 95% confidence interval (CI) 0.66–0.99; *p* = 0.041) [8]. Based on the results, single-agent eribulin has been approved for the treatment of previously treated mBC in the US, the EU, and Japan; in Japan, it can be used in any line of therapy for inoperable or mBC of any subtype.

### Study rationale

The combination of eribulin with anti-HER2 therapy has been studied in three phase II studies and may be a reasonable, potential option as a first-line treatment for HER2^+^ mBC. In one study, an ORR of 71.2% was reported after the administration of a median of 10 cycles of eribulin plus a median of 11 cycles of Tmab [9]. In another study, which was conducted in Japan (JBCRG-M03), eribulin, administered in combination with Tmab plus Pmab, was tolerated well until disease progression, with most patients achieving a > 90% relative dose intensity for eribulin for up to eight cycles. The triplet regimen produced an ORR of 87.5% and a median PFS of 20.5 months [10]. Furthermore, Inoue et al. recently reported a PFS of 23.1 months and an ORR of 80.0% in patients receiving eribulin in combination with Tmab plus Pmab as a first-line treatment for HER2^+^ mBC [11]. Although no data are available from head-to-head comparison of eribulin versus taxanes, existing phase II or phase III data comparing capecitabine with taxanes or eribulin [12, 13] suggest better QOL of mBC patients on eribulin compared with taxanes.

### Objectives

The objective of this phase III, non-inferiority study (JBCRG-M06) is to compare Tmab, Pmab, and eribulin versus Tmab, Pmab, and taxane (DTX or paclitaxel (PTX)) in efficacy (in terms of PFS), safety and QOL based on an overall review of the results of these studies together with the CLEOPATRA study.

## Methods/design

### Study design

**Fig. 1:**
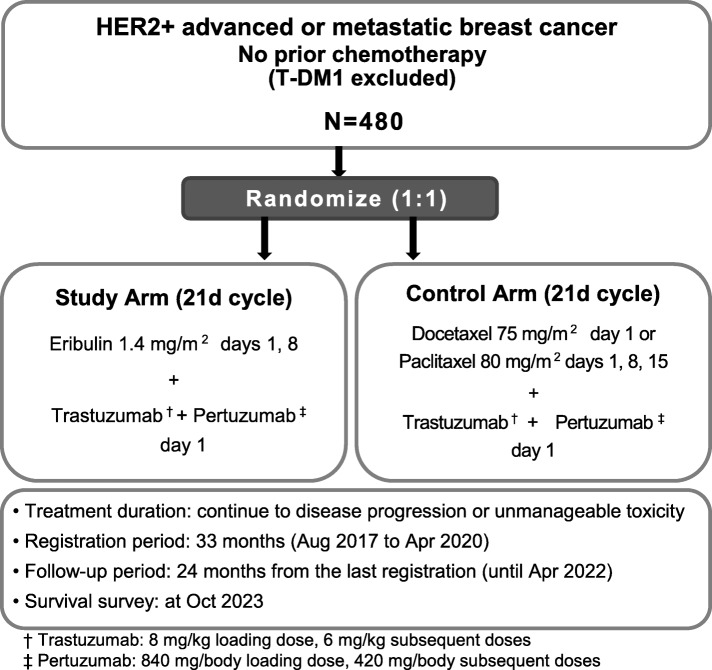
Study design flowchart

**Fig. 2:**
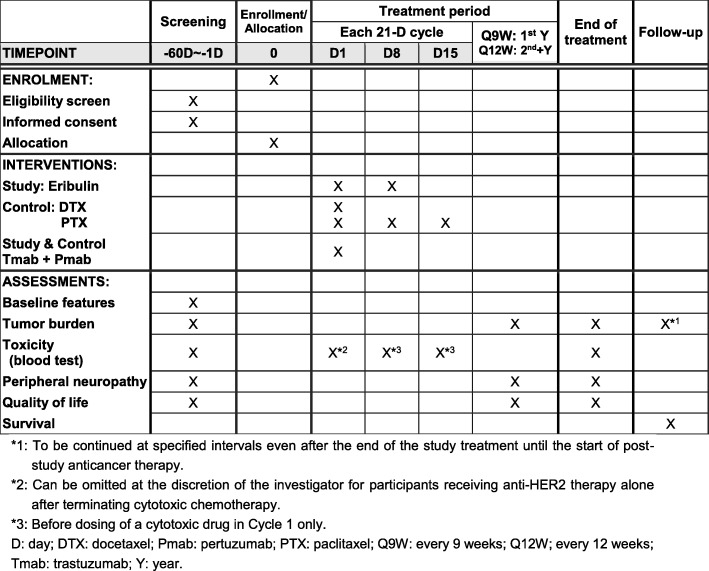
Schedule of enrollment, interventions, and assessments

This multicenter, randomized, open-label, parallel-group, phase III study is primarily designed to demonstrate the non-inferiority of Tmab, Pmab, and eribulin against Tmab, Pmab, and taxane in terms of PFS as a first-line or second-line treatment (following Tmab-emtansine (T-DM1)) for HER2^+^ mBC. The study also aims to show better QOL in patients treated with Tmab, Pmab, and eribulin in a secondary endpoint. The study design flowchart is shown in Fig. 1. This protocol was written following the Standard Protocol Items: Recommendations for Interventional Trials (SPIRIT) Checklist (see Additional file 1). The schedule of participant recruitment, interventions, and assessments is presented in Fig. 2 (the SPIRIT Checklist).

### Study setting

This study was commenced on 28 August 2017 and is recruiting participants at 136 Japanese oncology centers. The study is collaboratively performed by the Japan Breast Cancer Research Group (JBCRG; office@jbcrg.jp) and Eisai Co., Ltd. The JBCRG is to collect, assemble, analyze, and interpret data from the study independently of Eisai Co., Ltd.

### Outcome measures

The primary endpoint of the study is PFS as per investigator according to Revised Response Evaluation Criteria in Solid Tumors (RECIST) v1.1 criteria [14]. Key secondary endpoints include ORR, duration of response, OS, patient-reported outcomes (PROs; QOL and incidence of peripheral neuropathy), safety and biomarkers, new-metastasis-free survival (nMFS), and duration of post-study treatment. As a subsidiary study, translational research is also planned to identify biomarkers that could predict the response to treatment and help select patients most likely to benefit from individual treatments.

### Participants

The study participants will be women with pathologically diagnosed advanced/recurrent breast cancer positive for HER2 at the primary or metastatic site who meet the following eligibility criteria:

#### Inclusion criteria

- Age 20–70 years
- No prior cytotoxic chemotherapy (excluding T-DM1) for advanced/recurrent disease
- At least 6 months since prior neoadjuvant or adjuvant cytotoxic chemotherapy
- Presence of any radiologically evaluable lesion
- Left ventricular ejection fraction (LVEF) ≥ 50% at baseline
- Eastern Cooperative Oncology Group (ECOG) performance status (PS) score of 0 or 1
- Adequate organ function: neutrophil count ≥ 1500/mm^3^, platelet count ≥ 100,000/mm^3^, hemoglobin ≥ 9.0 g/dL, total bilirubin ≤ 1.5 mg/dL, AST (GOT), ALT (GPT) less than 100 IU (equal to or less than 150 IU in cases with liver metastasis), serum creatinine ≤ 1.5 mg/dL
- Life expectancy of at least 6 months
- Written informed consent
- Willingness to undergo QOL assessment

#### Key exclusion criteria

- Grade ≥ 2 peripheral neuropathy or any other Grade ≥ 3 non-hematologic toxicity (Japan Clinical Oncology Group (JCOG) version of the Common Terminology Criteria for Adverse Events (CTCAE) v4.0)
- Symptomatic or uncontrollable central nervous system metastasis
- Any other active malignancy, systemic infection or interstitial pneumonia
- Current uncontrolled hypertension or unstable angina, current or previous clinically significant cardiovascular illness, or a recent (≤ 6 months) history of myocardial infarction
- Major surgery or injury within 28 days of enrollment or surgery scheduled during the study
- Pregnancy or breastfeeding, or unwillingness to use effective contraception
- Allergy to Tmab and/or Pmab

### Ethics

All individuals involved in the study are to comply with the Declaration of Helsinki, the Clinical Trials Act, and the International Conference on Harmonization Good Clinical Practice (ICH-GCP) guideline. The study protocol was approved by the Certified Clinical Research Review Committee as well as the Institutional Review Board of each participating institution. The study was registered on 28 June 2017 with ClinicalTrials.gov (ID: NCT03264547) and the University Hospital Medical Information Network (UMIN) of Japan (protocol ID: 000027938). Further details of the protocol are available at the following URL: https://upload.umin.ac.jp/cgi-open-bin/ctr_view.cgi?recptno=R000031805. The investigators will obtain written informed consent from each participant before screening.

### Intervention

#### Description of components

Tmab and Pmab are two common components of the study and control treatments and will be administered every 3 weeks (on day 1) by intravenous infusion at a loading dose of 8 mg/kg followed by 6 mg/kg and at a loading dose of 840 mg followed by 420 mg, respectively. Participants in the study arm will additionally receive intravenously administered 1.4 mg/m^2^ eribulin on days 1 and 8 of each 3-week cycle. Those in the control arm will additionally receive intravenously administered 75 mg/m^2^ DTX on day 1 or 80 mg/m^2^ PTX on days 1, 8, and 15 of each 3-week cycle.

#### Dose modification

A Grade ≥ 2 hematologic or non-hematologic toxicity will require a dose delayed or skipped for a cytotoxic drug. A dose following a delayed or skipped dose will be reduced to the next lower level (1.1 or 0.7 mg/m^2^ for eribulin, 60 or 45 mg/m^2^ for DTX, and 60 or 50 mg/m^2^ for PTX). If the day-1 dose of a cytotoxic drug is delayed, the doses of the anti-HER2 drugs in this cycle will also be delayed. Failure to recover from toxicities even after a dose delay to the next cycle will lead to termination of the cytotoxic chemotherapy. In both arms, participants can continue to receive anti-HER2 therapy alone and remain in the study even after termination of cytotoxic chemotherapy. The administration of Tmab and Pmab will be delayed or canceled in the event of left ventricular dysfunction or infusion reaction. If the administration of the two anti-HER2 drugs is delayed for ≥ 6 weeks, the participant will then be discontinued from the protocol treatment and be regarded as being “censored.” Participants are supposed to remain on the protocol treatment until radiologic, cytologic or photographic evidence of disease progression as per investigator or the development of any unmanageable toxicity. Concomitant use of non-trial anticancer therapies is prohibited.

#### Randomization

Using a web-based interactive system, the Data Center of JBCRG will enroll consenting eligible patients and randomize them in a ratio of 1:1 to receive the study or control treatment. Randomization will be done by dynamic allocation simultaneously adjusted with the following minimization factors: (1) prior perioperative taxane use (two or more cycles of DTX or six or more weekly doses of PTX); (2) prior treatment with an anti-HER2-antibody-drug conjugate for recurrent disease; and (3) visceral metastasis at enrollment (yes or no). The Data Center will generate and securely keep the randomization schedule. The Data Center will document detailed methods of randomization in its written procedure and keep it unavailable to the investigators until completion of the study to minimize the predictability of a random sequence. Neither participants nor investigators will be blinded to the treatments assigned to individual participants.

#### Follow-up

Baseline evaluations are to be conducted at screening (within 14, 28 or 60 days before enrollment according to parameters). Baseline evaluations comprise medical history (present illness and prior treatments), physical examination (including ECOG PS), vital signs, electrocardiogram, echocardiographic or multigated acquisition (MUGA)-based LVEF measurement, radiologic tumor assessments (chest, abdomen, and brain (only if brain involvement is suspected by the investigator) computed tomography (CT)/magnetic resonance imaging (MRI) as well as bone scintigraphy), hematology, serum chemistry, tumor markers, and urine human chorionic gonadotropin (only for those with childbearing potential).

After the start of the protocol treatment, participants will be examined for vital signs, ECOG PS, hematology, and serum chemistry on day 1 of each 21-day cycle. To determine tumor response, participants will undergo radiologic tumor assessments and tumor-marker measurements every 9 weeks during the first year and every 12 weeks thereafter.

European Organization for Research and Treatment of Cancer (EORTC) Quality of Life Questionnaire (QLQ) Module C30 (EORTC QLQ-C30), breast cancer-specific EORTC QLQ-BR23, and the five-level version of the EuroQoL five-dimension (EQ-5D-5 L) questionnaires will be administered to the individual participants to assess their QOL. The PRO version of the CTCAE (PRO-CTCAE) will be used to assess the severity of peripheral neuropathy. These assessments will be done at baseline, every 9 weeks during the first year of the protocol treatment, and every 12 weeks thereafter.

Throughout the period of the protocol treatment, adverse events will be collected and followed up until resolved, normalized or returned to the baseline. Further follow-up will be made for adverse events considered related to the protocol treatment that persist at the end of the treatment.

All participants will be followed until December 2022, i.e., 2 years after the last participant recruitment. All participants who complete the protocol (excluding those who die during the study) will be examined for survival in June 2024.

#### Monitoring

To determine the suitability of continuing the study and the need for amending the protocol, an Independent Data Monitoring Committee (IDMC), which is independent of the sponsor and funder, will monitor the study progress, safety data, and key efficacy endpoints at appropriate frequencies.

All serious adverse events (SAEs) will be reported to the principal investigator, who will assess its expectedness and causal relationship with the protocol treatment and determine the need for suspending participant recruitment. If the SAE is considered related to the treatment, the principal investigator will inform all investigators via the JBCRG administrative office. The principal investigator can consult the IDMC about the assessment of the SAE, the suitability of continuing the study, and the need for amending the protocol.

#### Sample size

A sample size of 480 (240 per arm) is based on the primary endpoint PFS. Based on the CLEOPATRA study [15], it was estimated that 50% of the cases will be registered in the study at stage 4 (inoperable breast cancer) (expected PFS of 18 months) and 50% of the cases will be registered in the study after relapse following the preoperative and postoperative treatment including postoperative trastuzumab (expected PFS of 16 months). The expected PFS for secondary treatment in HER2-positive patients who have previously undergone T-DM1 was estimated to be 11 months, based on the PFS of pertuzumab + trastuzumab + capecitabine in the PHEREXA study for secondary treatment [16], which was 11.1 months.

Based on existing data and an expected 6:4 ratio of first-line versus second-line patients enrolled in the study, a median PFS of 14.2 months is expected in the control arm. Assuming a two-sided type-I error of 0.05 and a statistical power of 80%, at least 387 PFS events are needed to show non-inferiority of the study arm against the control arm (expected HR, 1.00) with a non-inferiority margin (HR_0_) of 1.33. To observe this number of events, at least 456 participants need to be enrolled during the planned period (2 years and 9 months) and to be followed up until 2 years after last participant enrollment. The planned sample size (*n* = 480) further takes account of the expected number of participants disqualified or participants who drop out after enrollment.

#### Interim analysis

The interim analysis will be performed when 80 PFS events have occurred, and the study will be discontinued on the basis of unprofitability if the probability of the study group (combination of trastuzumab, pertuzumab, and eribulin) in achieving the non-inferiority result of 1.33 to the control group is considered low (5% or lower).

### Analysis methods

#### Primary endpoint

The primary analysis set for PFS will be the intent-to-treat (ITT) population. From Kaplan-Meier estimates of PFS, median PFS and its 95% CI will be calculated for each arm. A Cox proportional hazard model which includes the three minimization factors as covariates will be used to estimate the HR for PFS events in the study arm versus the control arm with its two-sided 95% CI.

Non-inferiority testing will be performed using two non-inferiority margins. First, the non-inferiority of the study arm will be tested with non-inferiority margins of 1.33. If the upper bound of the 95% CI of HR is less than 1.33, non-inferiority will then be tested with an HR_0_ of 1.25. If the upper bound of the 95% CI of HR is less than 1.25, non-inferiority of the study arm will be claimed with this more conservative criterion, and its superiority over the control arm will then be tested. If the upper bound of the 95% CI of HR is less than 0, superiority of the study arm will be claimed.

#### Secondary endpoints

For time-to-event data (duration of response, OS, and nMFS), survival curves will be estimated using the Kaplan-Meier method and compared between the two arms using the log-rank test in an exploratory manner. For ORR, the point estimate of the between-arm difference and the one-sided 95% CI for the point estimate will be calculated. If required, ORR data will be compared between the two arms using the chi-square test.

Impacts of treatments on QOL will be evaluated and compared based on a minimally important difference (MID) of EORTC QLQ-C30 Global Health Status (GHS) score of 10 points. A ≥ 10-point decrease of GHS score from baseline is defined as a clinically significant QOL deterioration event (QOL deterioration). At each specified time, the proportions of participants with and without QOL deterioration will be calculated. Using the Kaplan-Meier method, the cumulative rate of QOL deterioration up to 1 year after randomization will be estimated for each arm. The point estimate of the between-arm difference and the 95% CI for the point estimate will be calculated.

Adverse events will be graded according to the CTCAE v4.0-JCOG and the PRO-CTCAE, and their frequencies by grade will be calculated for each arm. The incidence rates of Grade ≥ 3 events will be compared between the two arms. Events of special interest will include neurotoxicity (peripheral neuropathy) and cardiotoxicity (congestive heart failure, LVEF < 40% and a > 10% reduction of LVEF from baseline). Imputation of missing data has not been planned.

### Completion and premature termination of the clinical study

#### Discontinuation of the study

In the event that it becomes necessary to discontinue the study prematurely due to reasons such as recommendation by the Independent Data Monitoring Committee, the principal investigator must immediately report the discontinuation of the study and the reason for this to the investigators at each study site.

An interim analysis will be performed. The study will be discontinued if the continuation of the study is determined unprofitable.

### Dissemination

The results of this study will be reported in accordance with the international Consolidated Standards of Reporting Trials (CONSORT) Statement. Findings from the study will be submitted for publication in peer-reviewed journals and/or presented at international breast cancer conferences, regardless of whether the primary endpoint of the study is met or not. The authors will be individuals who have made substantial contributions to the design and conduct of the study.

## Discussion

Breast cancer has placed an increasing burden on patients and health care systems worldwide. The prognosis of breast cancer diagnosed at the early stages has improved considerably due to the greater use of multidisciplinary care and the development of innovative modalities of systemic treatment in recent decades. Although patients with mBC still have a poor prognosis and limited treatment options with curative intent, the current status of developing new drugs and new combinations for advanced-stage disease suggests that mBC patients will have improved outcomes and an increased probability of being cured in the near future.

To the best of our knowledge, this is the only confirmatory study that aims to establish a less toxic alternative to taxanes to be combined with double blockade of HER2 with Tmab and Pmab for HER2^+^ breast cancer in the advanced-disease setting. In the adjuvant and neoadjuvant settings, ongoing studies are comparing Tmab, Pmab, and taxane with T-MD1 plus Pmab (NCT01966471) and with Tmab, Pmab, and endocrine therapy (NCT03272477), respectively. If the present study shows the non-inferiority of Tmab, Pmab, and eribulin against Tmab, Pmab, and taxane with an HR_0_ of 1.25, Tmab, Pmab, and eribulin may be recommended as a standard first-line or second-line treatment option for HER2^+^ mBC along with the current standard. If its superiority over the current standard is further demonstrated, it may become a new standard first-line regimen for HER2^+^ mBC. Even if its non-inferiority is only shown with non-inferiority margins of 1.33, it may become a treatment option that may even be considered as recommended or standard, if treatment with this regimen results in better QOL or has an improved toxicity profile. Thus, if the primary endpoint of this study is met, Tmab, Pmab, and eribulin may become a useful addition to the armamentarium for oncologists treating patients with HER2^+^ mBC.

### Trial status

This study opened for recruitment in August 2017, with recruitment expected to be completed by December 2020. The first patient was enrolled in October 2017, and the actual number of patients recruited as of 31 January 2020 was 244.

The protocol version 1.1 was approved on 12 October 2018. The major protocol amendments made up to this version include adding a test for superiority of the study arm over the control arm if the non-inferiority of the study arm is shown to have a margin of 1.25. This amendment was based on the finding from a single-arm, open-label, phase II study [10] that suggests the potential achievement of a longer PFS in the study arm than in the control arm.

The current protocol is version 2.0 and was approved on 5 November 2019. Because of delayed registration, the registration and follow-up period for OS will be until December 2020 and June 2024, respectively.

## Supplementary information

**Additional file 1.** Standard Protocol Items: Recommendations for Interventional Trials (SPIRIT) 2013 Checklist: recommended items to address in a clinical trial protocol and related documents.

## Data Availability

Not applicable

## Abbreviations

CI: Confidence interval
CT: Computed tomography
CTCAE: Common Terminology Criteria for Adverse Events
DTX: Docetaxel
ECOG: Eastern Cooperative Oncology Group
EORTC QLQ-C30: European Organization for Research and Treatment of Cancer Quality of Life Questionnaire Module C30
GHS: Global Health Status
HER2: Human epidermal growth factor receptor 2
HR: Hazard ratio
ICH-GCP: International Conference on Harmonization Good Clinical Practice
ITT: Intent-to-treat
JBCRG: Japan Breast Cancer Research Group
JCOG: Japan Clinical Oncology Group
LVEF: Left ventricular ejection fraction
mBC: Metastatic breast cancer
MRI: Magnetic resonance imaging
MUGA: Multigated acquisition
nMFS: New-metastasis-free survival
ORR: Objective response rate
OS: Overall survival
PFS: Progression-free survival
Pmab: Pertuzumab
PRO: Patient-reported outcome
PRO-CTCAE: Patient-Reported Outcomes version of the Common Terminology Criteria for Adverse Events
PS: Performance status
PTX: Paclitaxel
RECIST: Response Evaluation Criteria in Solid Tumors
SAE: Serious adverse event
T-DM1: Trastuzumab-emtansine
Tmab: Trastuzumab
UMIN: University Hospital Medical Information Network
