# Correction to: Trastuzumab, pertuzumab, and eribulin mesylate versus trastuzumab, pertuzumab, and a taxane as a first-line or second-line treatment for HER2-positive, locally advanced or metastatic breast cancer: study protocol for a randomized controlled, non-inferiority, phase III trial in Japan (JBCRG-M06/EMERALD)

**DOI:** 10.1186/s13063-020-04408-w

**Published:** 2020-06-08

**Authors:** Toshinari Yamashita, Norikazu Masuda, Shigehira Saji, Kazuhiro Araki, Yoshinori Ito, Toshimi Takano, Masato Takahashi, Junji Tsurutani, Kei Koizumi, Masahiro Kitada, Yasuyuki Kojima, Yasuaki Sagara, Hiroshi Tada, Tsutomu Iwasa, Takayuki Kadoya, Tsuguo Iwatani, Hiroki Hasegawa, Satoshi Morita, Shinji Ohno

**Affiliations:** 1 Department of Breast Surgery, Kanagawa Cancer Center, 2-3-2 Nakao, Asahi-ku, Yokohama-shi, Kanagawa 241-8515 Japan; 2 Department of Surgery, Breast Oncology, National Hospital Organization Osaka National Hospital, 2-1-14 Hoenzaka, Chuou-ku, Osaka, 540-0006 Japan; 3 Department of Medical Oncology, Fukeushima Medical University, 1 Hikarigaoka Fukushima, Fukushima, 960-1295 Japan; 4 Department of Breast Surgery, Gunma Prefectural Cancer Center, 617-1 Takahayashinishicho, Ota, Gunma 373-8550 Japan; 5 Breast Medical Oncology, Breast Oncology Center, The Cancer Institute Hospital of JFCR, 3-8-31 Ariake Koto-ku, Tokyo, 135-8550 Japan; 6 Department of Medical Oncology, Toranomon Hospital, 2-2-2 Toranomon, Minato-ku, Tokyo, 105-8470 Japan; 7 Breast Surgery, NHO Hokkaido Cancer Center, 2-3-54 Yonjyo Kikusui Shiraishi-ku, Sapporo-shi, Hokkaido 003-0804 Japan; 8 Department of Medical Oncology, Showa University Hospital, 1-5-8 Hatanodai Shinagawa-ku, Tokyo, 142-8666 Japan; 9 First Department of Surgery, Hamamatsu University School of Medicine, 1-20-1 Handayama, Higashi-ku, Hamamatsu City, Shizuoka 431-3192 Japan; 10 Breast Disease Center, Asahikawa Medical University Hospital, 1-1 Higashi 2-jyo 1-chome, Midorigaoka, Asahikawa-shi, Hokkaido 078-8510 Japan; 11 Department of Breast Surgery, St. Marianna University School of Medicine Hospital, 2-16-1 Sugao Miyamae-ku, Kawasaki-shi, Kanagawa 216-8511 Japan; 12 Breast Surgical Oncology, Sagara Hospital, 3-31 Matsubaracho Kagoshima-shi, Kagoshima, 892-0833 Japan; 13 Department of Breast and Endocrine Surgical Oncology, Tohoku University Hospital, 1-1 Seiryocho Aoba-ku, Sendai-shi, Miyagi 980-8574 Japan; 14 Oncology Internal Medicine, Kindai University Hospital, 377-2 Ohnohigashi Sayama-shi Osaka, Osaka, 589-8511 Japan; 15 Breast Surgery, Hiroshima University Hospital, 1-2-3 Kasumi Minami-ku Hiroshima-shi, Hiroshima, 734-8551 Japan; 16 Department of Breast Surgery, National Cancer Center Hospital East, 6-5-1 Kashiwanoha, Kashiwa, Chiba 277-8577 Japan; 17 Eisai Co., Ltd., 4-6-10 Koishikawa Bunkyo-ku, Tokyo, 112-8088 Japan; 18 Department of Biomedical Statistics and Bioinformatics, Graduate School of Medicine Kyoto University, 54 Kawaharacho, Shogoin, Sakyo-ku, Kyoto, 606-8507 Japan; 19 Breast Oncology Center, The Cancer Institute Hospital of JFCR, 3-8-31 Ariake Koto-ku, Tokyo, 135-8550 Japan

**Correction to: Trials (2020) 21:391**

**https://doi.org/10.1186/s13063-020-04341-y**

Following publication of the original article [1], the authors identified an error in Fig. 1, due to the change of the study protocol version (from 1.1 to 2.0). The following points in Fig. 1 need to be changed:

- The end of the registration period will be extended from April 2020 to December 2020.

- The duration of registration period will be changed from 33 to 41 months.

- The end of the follow-up period will be extended from April 2022 to December 2022.

- The end of the survival survey will be extended from October 2023 to June 2024.

The same changes apply to the text as well, as follows:

**Trial Status section**

- This study opened for recruitment in August 2017, with recruitment expected to be completed by December 2020. The first patient was enrolled in October 2017, and the actual number of patients recruited as of January 31, 2020 was 244.

- The current protocol is version 2.0 and was approved on November 5, 2019. Because of delayed registration, the registration and follow-up period for OS will be until December 2020 and June 2024., respectively.

**Follow-up section**

- All participants will be followed up until December 2022, i.e., 2 years after the last participant recruitment.

The correct Fig. 1 is presented below:

**Fig. 1:**
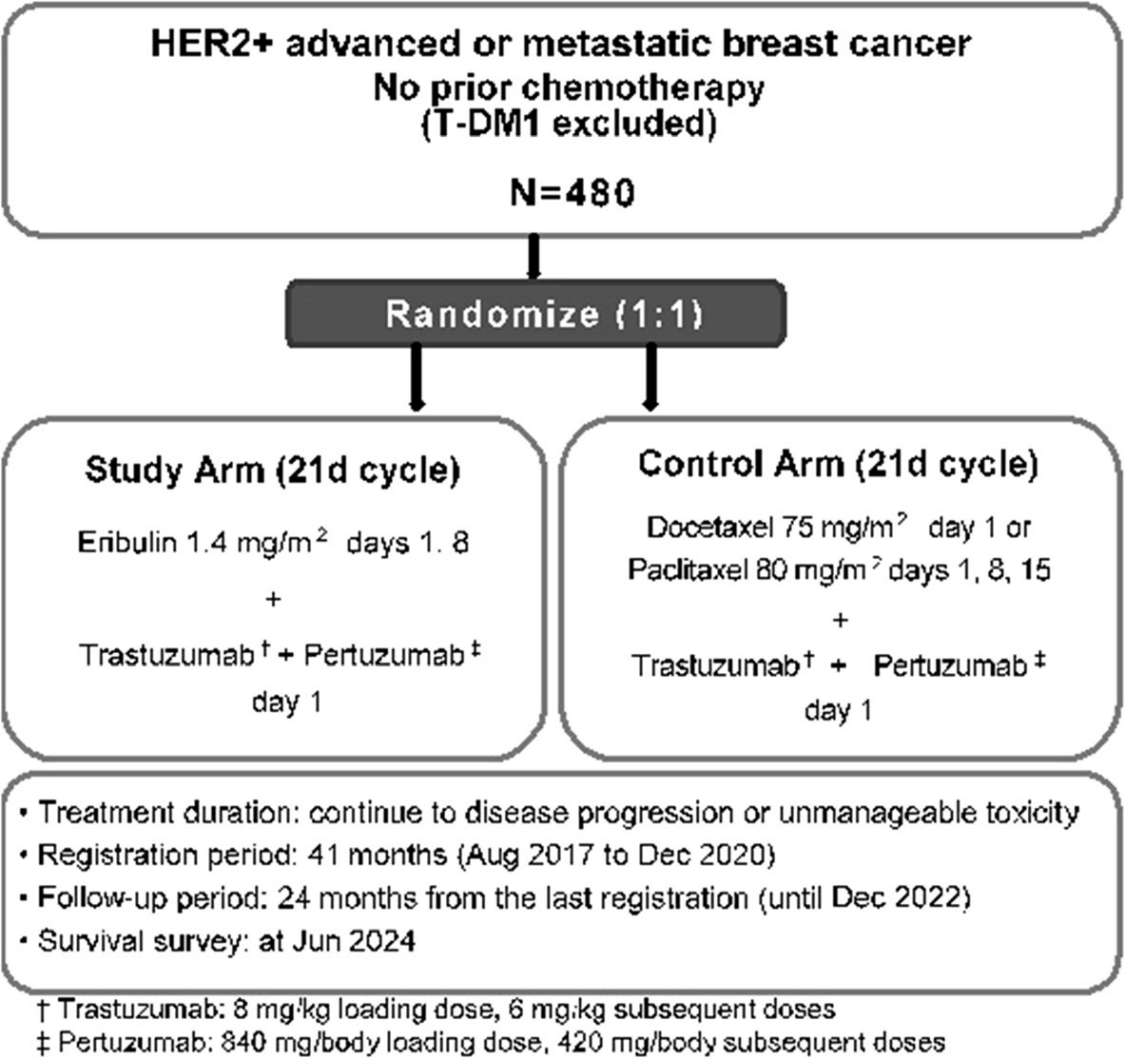
Study design flowchart
